# Appropriately designed studies are required before valproic acid, carbamazepine, and oxcarbazepine can be blamed for thyroid dysfunction

**DOI:** 10.1590/1806-9282.20250205

**Published:** 2025-08-08

**Authors:** Fulvio Alexandre Scorza, Carla Alexandra Scorza, Josef Finsterer

**Affiliations:** 1 Universidade Federal de São Paulo, Escola Paulista de Medicina – São Paulo (SP), Brazil.; 2 Neurology and Neurophysiology Center, Department of Neurology – Vienna, Austria.

Dear Editor,

We read with interest the article by Güngör et al. on the effect of antiseizure medication (ASM) on thyroid hormone parameters in children with cryptogenic epilepsy^1^. Thyroid-stimulating hormone (TSH) levels increased in patients receiving valproic acid (VPA) monotherapy compared to controls and pretreatment levels^1^. In patients receiving carbamazepine (CBZ) or oxcarbazepine (OXC) monotherapy, thyroxine-4 levels were lower compared to controls and before treatment, without any clinical effect^1^. It was concluded that changes in thyroid hormone levels by VPA, CBZ, and OXC had no influence on clinical presentation^1^. The study is noteworthy, but several points should be discussed.

The first point refers to the retrospective design of the study^1^. Retrospective designs have several disadvantages^2^. A retrospective design allows only limited control over the sampling of the population and only limited control over the type and quality of the predictor variables. In addition, the relevant predictors may not have been recorded in the medical record, and it may be difficult or impossible to establish confounding variables and causality. It is also inevitable that some information will be missing, as the data are based on the review of medical records that were not originally intended for the collection of data for research purposes. Selection and recall errors also affect the results, and the reasons for differences in the number of lost to follow-ups often cannot be determined, which can lead to bias^2^.

The second point relates to cerebral imaging. Although it was stated that all included patients had normal cerebral imaging, it was not stated whether they had had computed tomography or magnetic resonance imaging (MRI). Assuming that some of the patients had a cerebral MRI, it should have been specified what slice thickness they had and whether the scanner had a power of 1.5, 3.0, or 7.5 Tesla. The higher the resolution of an imaging technique, the more likely it is that structural or morphological abnormalities can be found. CCTs are generally not suitable for detecting hypomyelination, hamartomas, or gyration abnormalities.

The third point is that the family history of the included patients was not reported. In how many of the included patients was the family history positive for epilepsy, thyroid dysfunction, or other significant diseases associated with epilepsy? This is important because a number of patients with cryptogenic epilepsy may have genetic epilepsy.

The fourth point is that the ASM dosages varied among the included patients. Considering that the effect of ASM on thyroid function could be dose-dependent, it is conceivable that the results were skewed by the different doses taken by the patients in the study cohort.

The fifth point is that there is no mention of how many of the patients were SARS-CoV-2 positive and how many were not. As the data was collected between 2019 and 2024^1^, it is conceivable that at least some were SARS-CoV-2 positive at the time of blood sampling and that some of the patients with COVID-19 had thyroid dysfunction. Thyroid dysfunction is a known complication of SARS-CoV-2 infections^3^. SARS-CoV-2 has been found to cause thyroiditis in particular^4^. The influence of SARS-CoV-2 vaccination (SC2V) on thyroid function should also be investigated, as SC2V can be complicated by thyroid inflammation^5^.

Overall, this interesting study has shortcomings that relativize the results and their interpretation. Addressing these limitations could strengthen the conclusions and corroborate the study's message. Before concluding that VPA increases thyroid function and CBZ and OXC decrease it, homogenized and matched groups need to be analyzed.

## Data Availability

The datasets generated and/or analyzed during the current study are available from the corresponding author upon reasonable request.
